# Supplementary material for: Rethinking Sesquiterpenoids: A Widespread Hormone in Animals
Source: Int J Mol Sci. 2022 May 26;23(11):5998. doi: 10.3390/ijms23115998 (PMC9181382; doi:10.3390/ijms23115998)
Supplement: Supplementary file 1 [file ijms-23-05998-s001.zip › ijms- 1702691 suppl. materials/ijms- 1702691-Supplementary Figures.pdf]

Supplementary Figures

Rethinking sesquiterpenoids: A widespread hormone in animals

So and Kai et al

Table of contents

Figure S1. Phylogenetic tree of acetyl-CoA C-acetyltransferase (ACAT)

Figure S2. Phylogenetic tree of hydroxymethylglutaryl-CoA synthase (HMGCS)

Figure S3. Phylogenetic tree of hydroxymethylglutaryl-CoA reductase (HMGCR)

Figure S4. Phylogenetic tree of mevalonate kinase (MVK) and phosphomevalonate kinase (PMVK)

Figure S5. Phylogenetic tree of diphosphomevalonate decarboxylase (DPMD)

Figure S6. Phylogenetic tree of isopentenyl-diphosphate delta-isomerase (IPPI)

Figure S7. Phylogenetic tree of farnesyl pyrophosphate synthase (FPPS)

Figure S8. Phylogenetic tree of farnesyltransferase beta (FNTB)

Figure S9. Phylogenetic tree of ste24 endopeptidase (ste24)

Figure S10. Phylogenetic tree of protein-S-isoprenylcysteine O-methyltransferase (ICMT)

Figure S11. Phylogenetic tree of prenylcysteine oxidase (PCYOX)

Figure S12. Phylogenetic tree of aldehyde dehydrogenase 3 (ALDHIII)

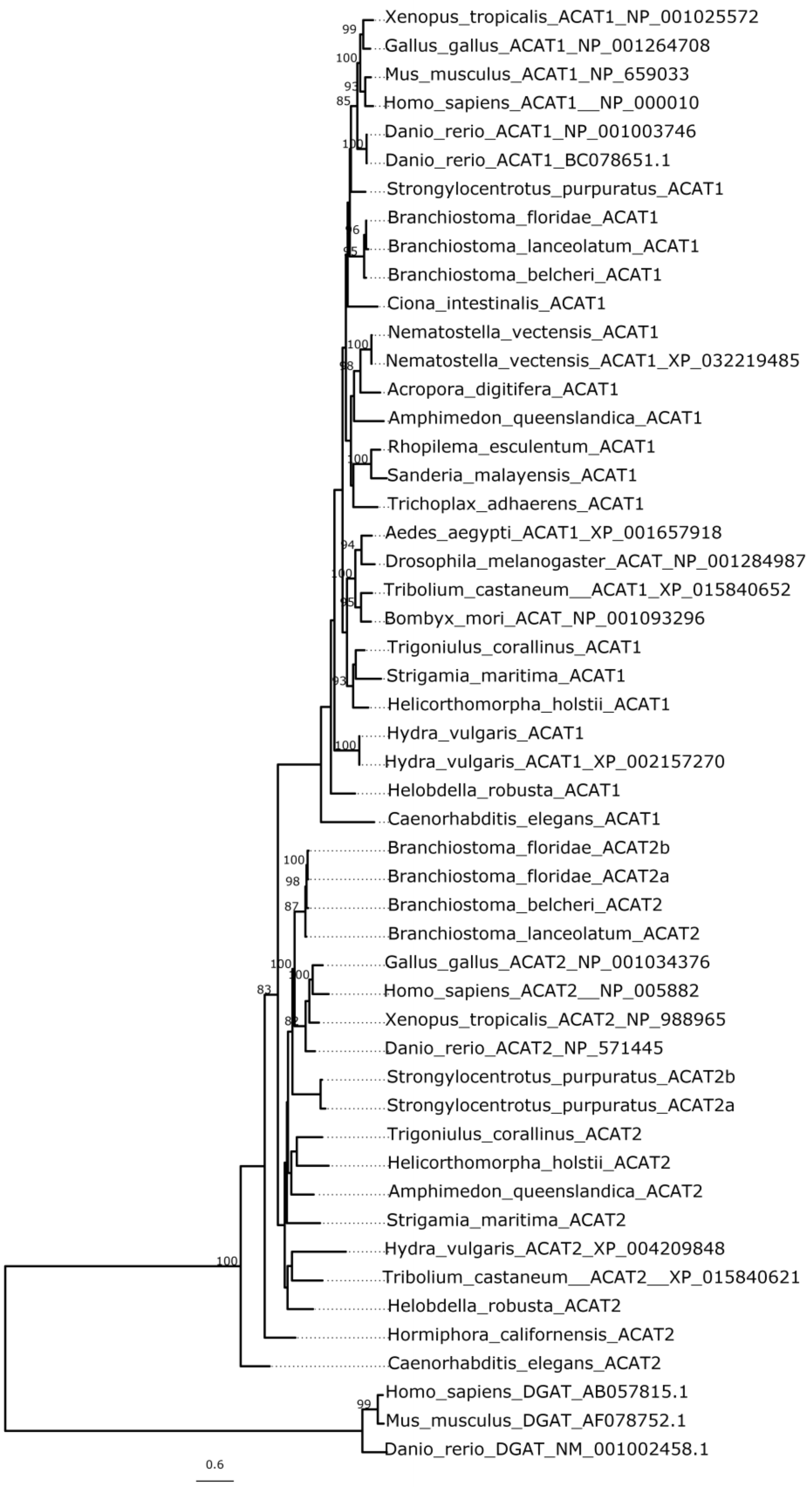

Supplementary Figure S1. Phylogenetic tree of acetyl-CoA C-acetyltransferase (ACAT). Tree is constructed with the maximum likelihood (ML) method (LG+G4) with 1000 bootstrap replicates. Only bootstrap values larger than 80% are indicated for clarity. Tree rooted using metazoan diglyceride acyltransferase (DGAT).

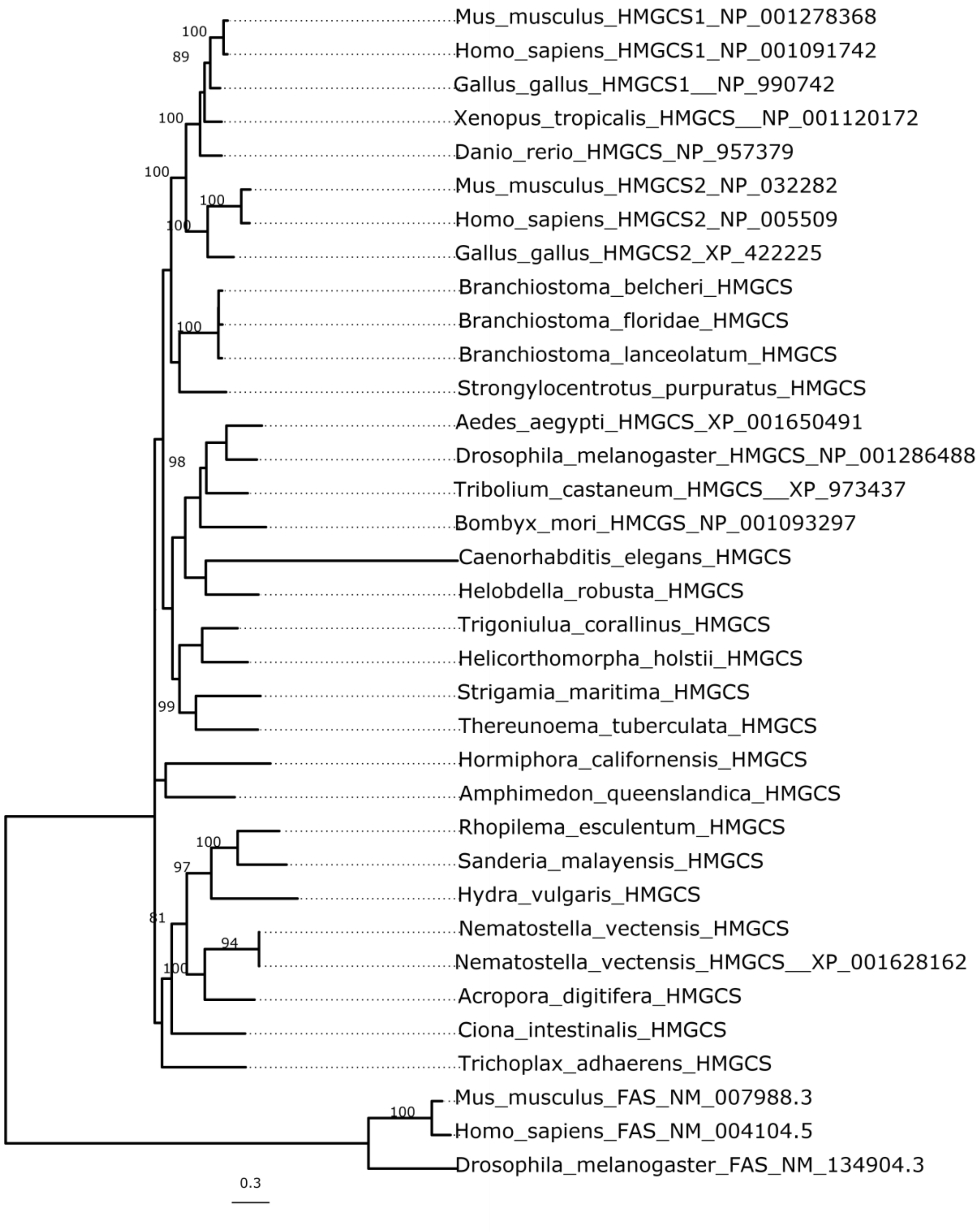

Supplementary Figure S2. Phylogenetic tree of hydroxymethylglutaryl-CoA synthase (HMGCS). Tree is constructed with the maximum likelihood (ML) method (LG+G4) with 1000 bootstrap replicates. Only bootstrap values larger than 80% are indicated for clarity. Tree rooted using metazoan fatty acid synthase (FAS).

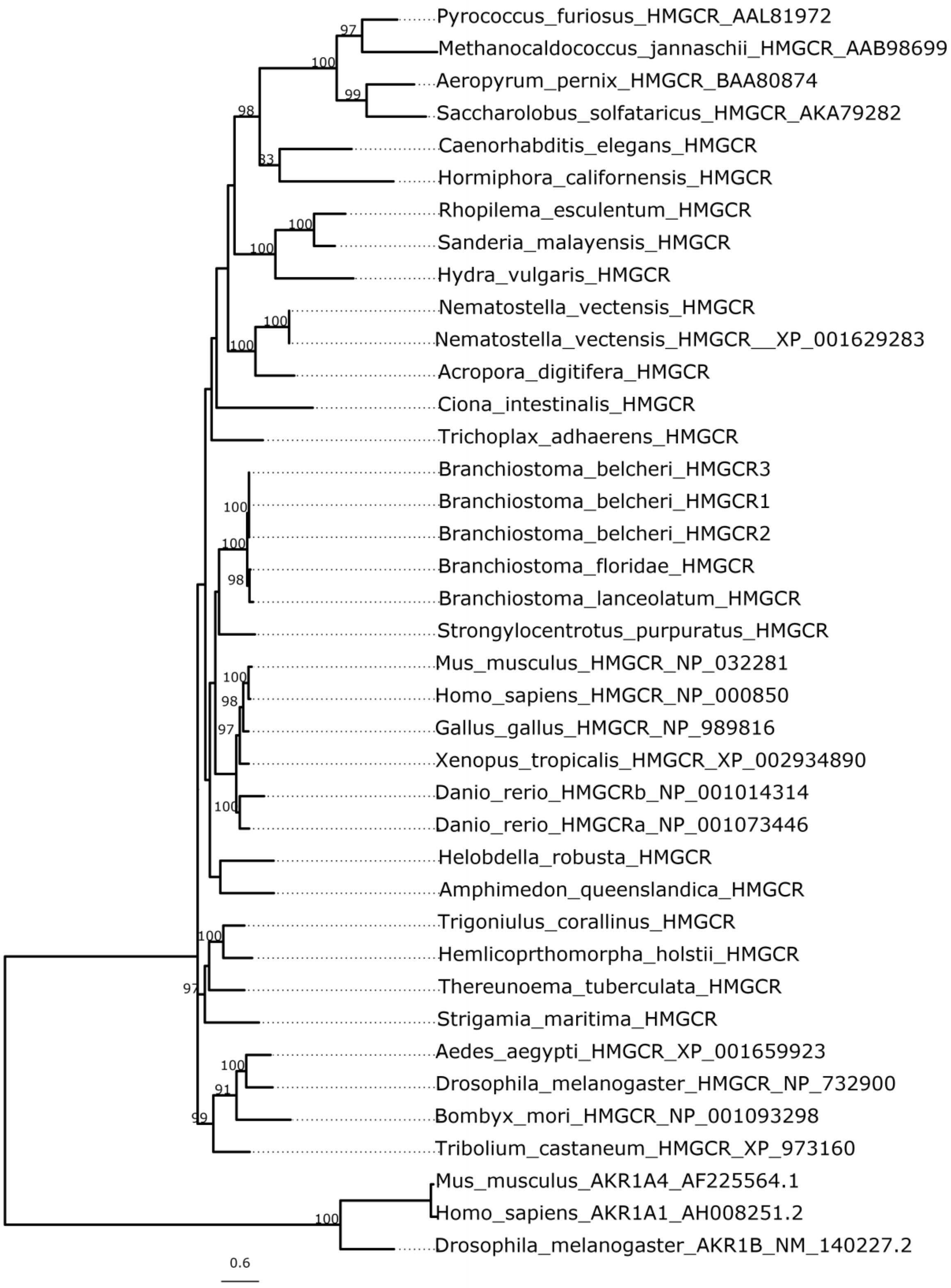

Supplementary Figure S3. Phylogenetic tree of hydroxymethylglutaryl-CoA reductase (HMGCR). Tree is constructed with the maximum likelihood (ML) method (LG+R5) with 1000 bootstrap replicates. Only bootstrap values larger than 80% are indicated for clarity. Tree rooted using metazoan aldo-keto reductase (AKR1).

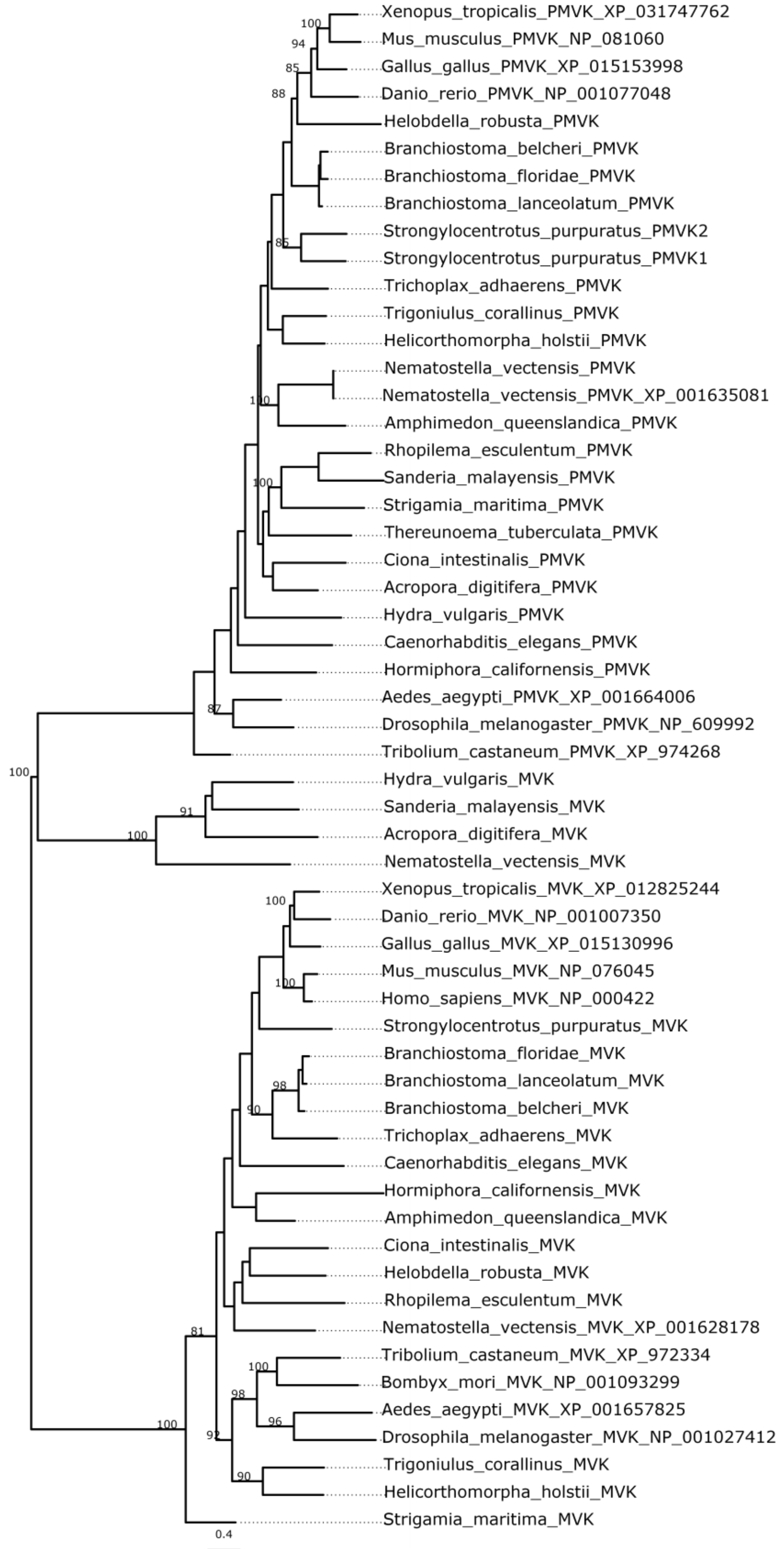

Supplementary Figure S4. Phylogenetic tree of mevalonate kinase (MVK) and phosphomevalonate kinase (PMVK). Tree is constructed with the maximum likelihood (ML) method (LG+R4) with 1000 bootstrap replicates. Only bootstrap values larger than 80% are indicated for clarity.

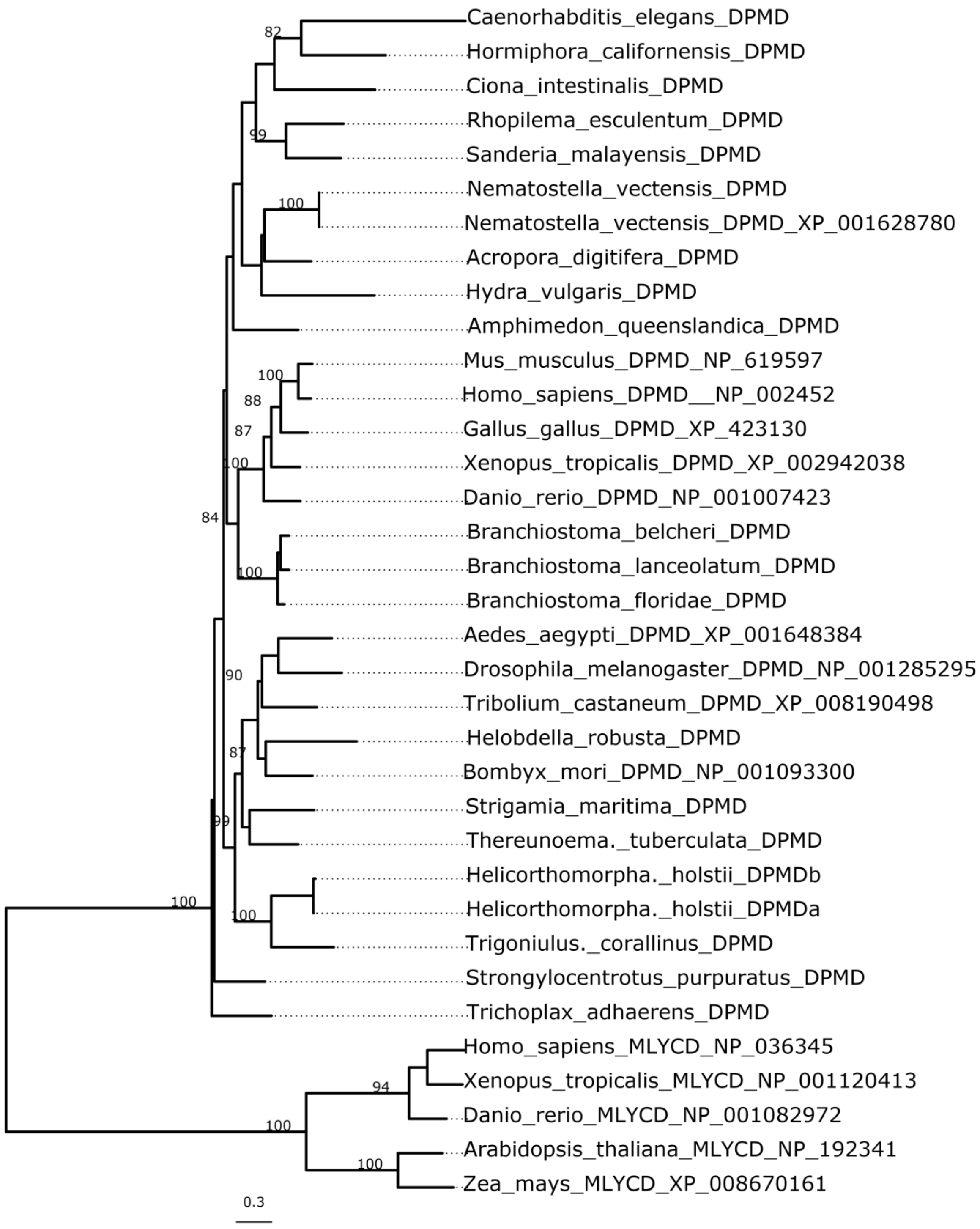

Supplementary Figure S5. Phylogenetic tree of diphosphomevalonate decarboxylase (DPMD). Tree is constructed with the maximum likelihood (ML) method (LG+G4) with 1000 bootstrap replicates. Only bootstrap values larger than 80% are indicated for clarity. Tree rooted using malonyl-CoA decarboxylase (MLYCD).

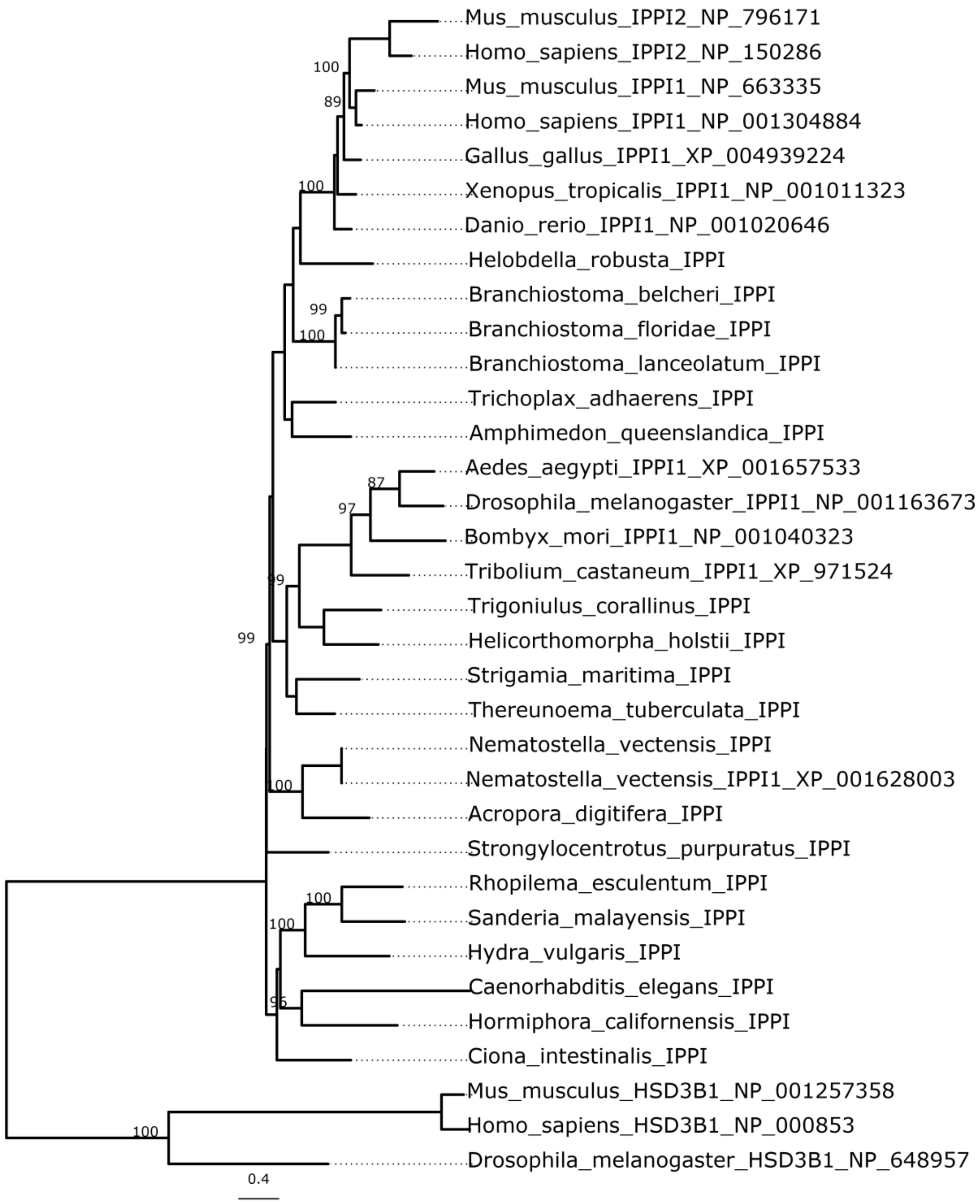

Supplementary Figure S6. Phylogenetic tree of isopentenyl-diphosphate delta-isomerase (IPPI). Tree is constructed with the maximum likelihood (ML) method (LG+I+G4) with 1000 bootstrap replicates. Only bootstrap values larger than 80% are indicated for clarity. Tree rooted using metazoan hydroxy-delta-5-steroid dehydrogenase, 3 beta- and steroid delta-isomerase 1 (HSD3B1).

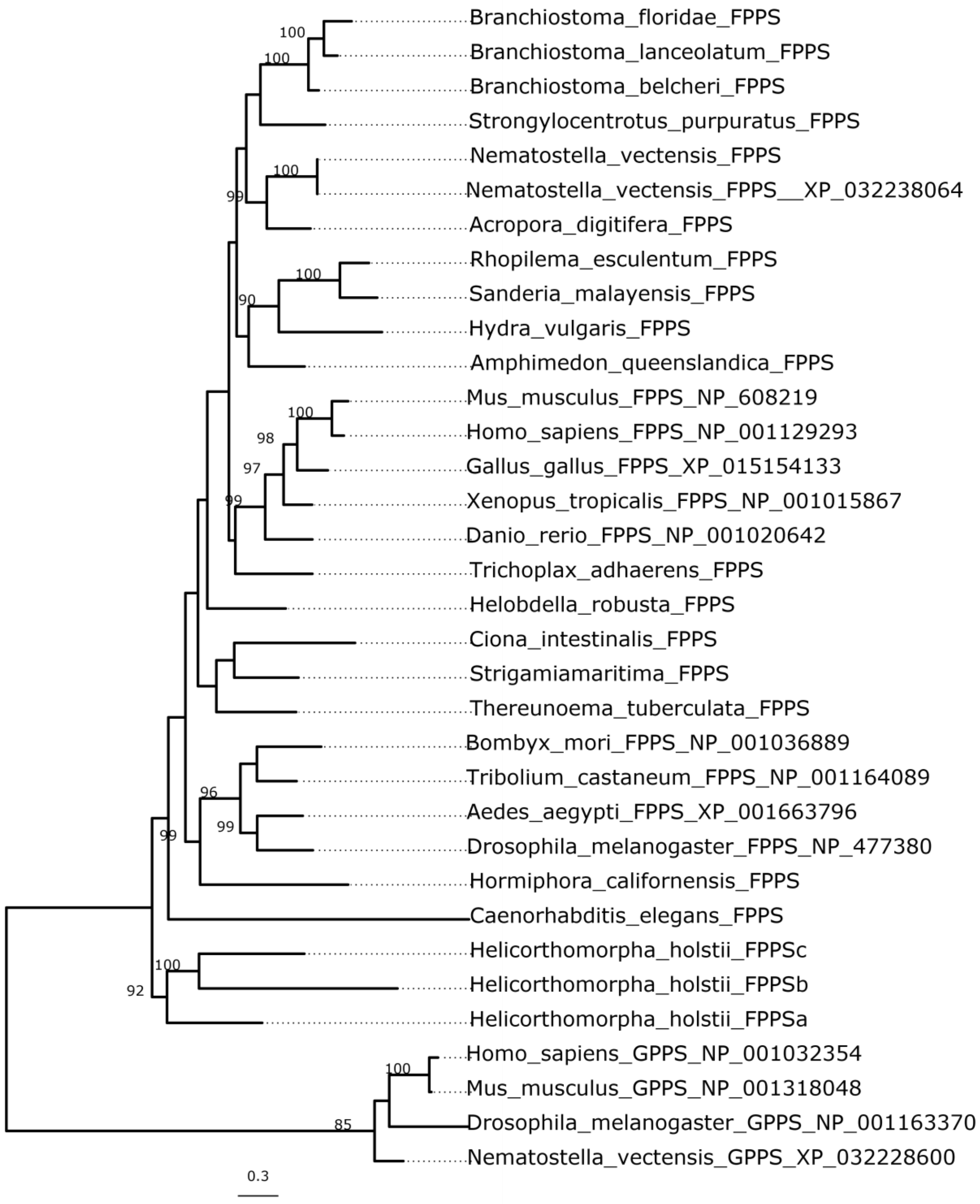

Supplementary Figure S7. Phylogenetic tree of farnesyl pyrophosphate synthase (FPPS). Tree is constructed with the maximum likelihood (ML) method (LG+I+G4) with 1000 bootstrap replicates. Only bootstrap values larger than 80% are indicated for clarity. Tree rooted using metazoan geranyl diphosphate synthase (GPPS).

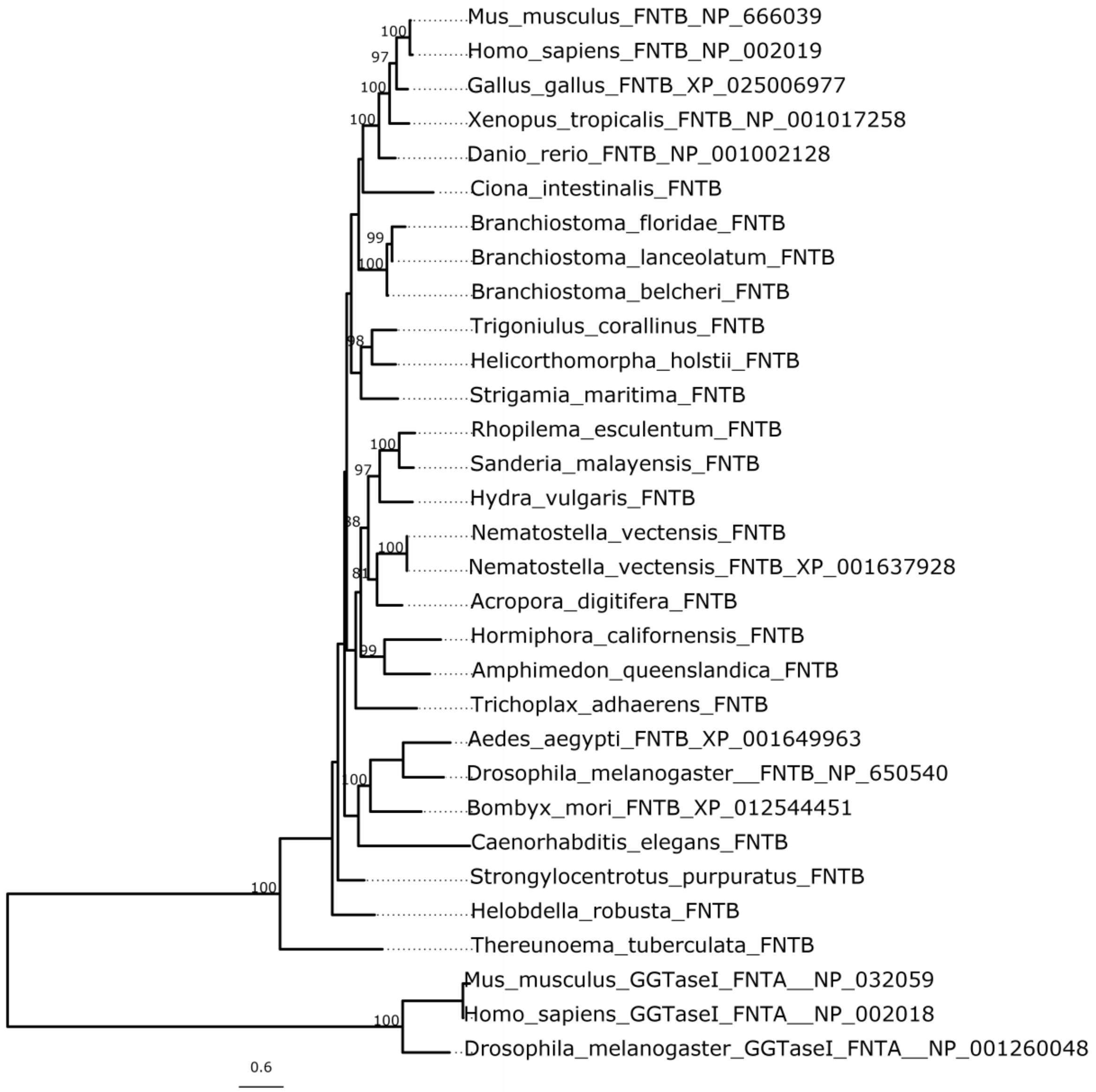

Supplementary Figure S8. Phylogenetic tree of farnesyltransferase beta (FNTB). Tree is constructed with the maximum likelihood (ML) method (LG+G4) with 1000 bootstrap replicates. Only bootstrap values larger than 80% are indicated for clarity. Tree rooted using metazoan geranylgeranyltransferase (FNTA).

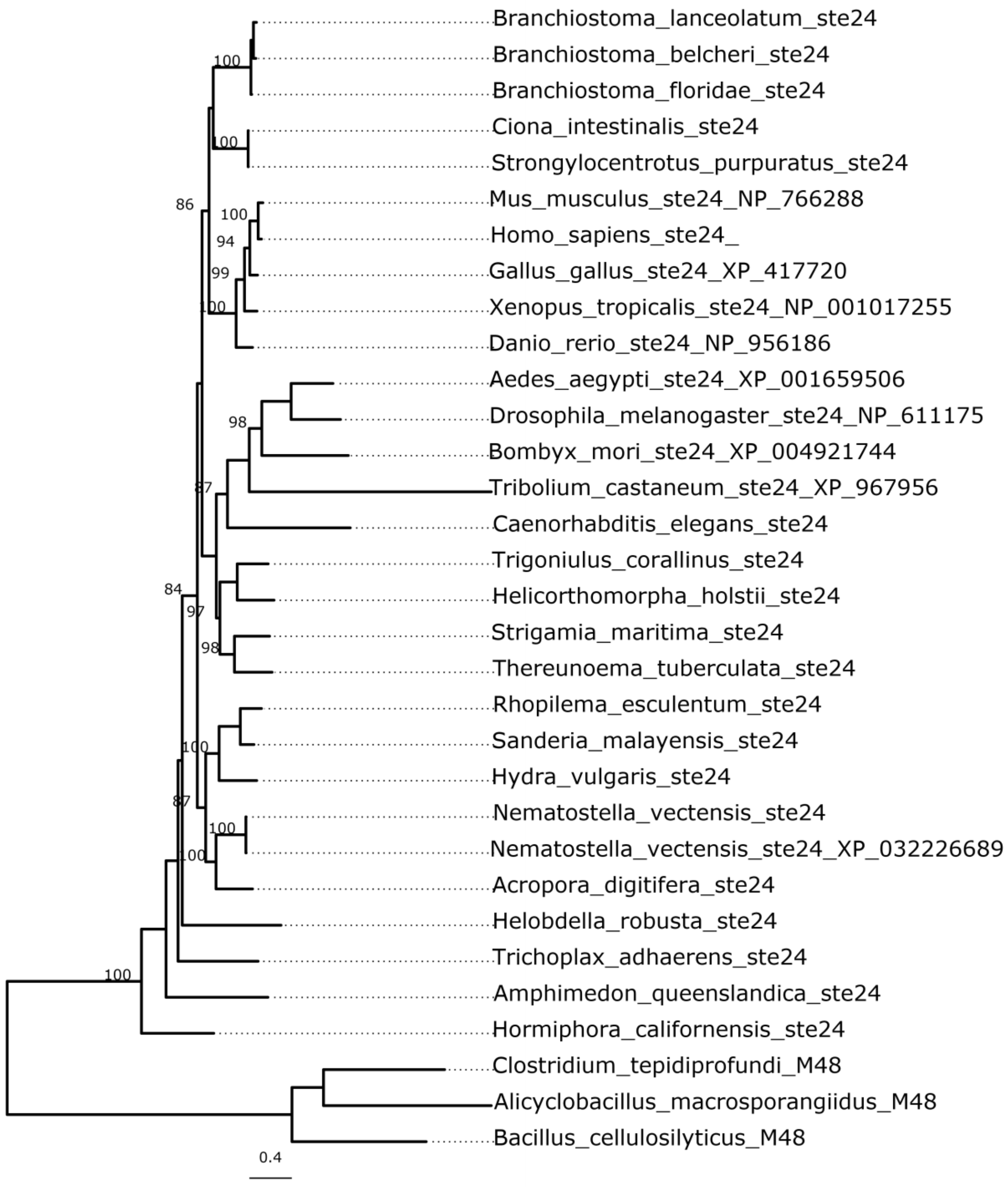

Supplementary Figure S9. Phylogenetic tree of ste24 endopeptidase (ste24). Tree is constructed with the maximum likelihood (ML) method (LG+G4) with 1000 bootstrap replicates. Only bootstrap values larger than 80% are indicated for clarity. Tree rooted using protozoan M48 metalloprotease.

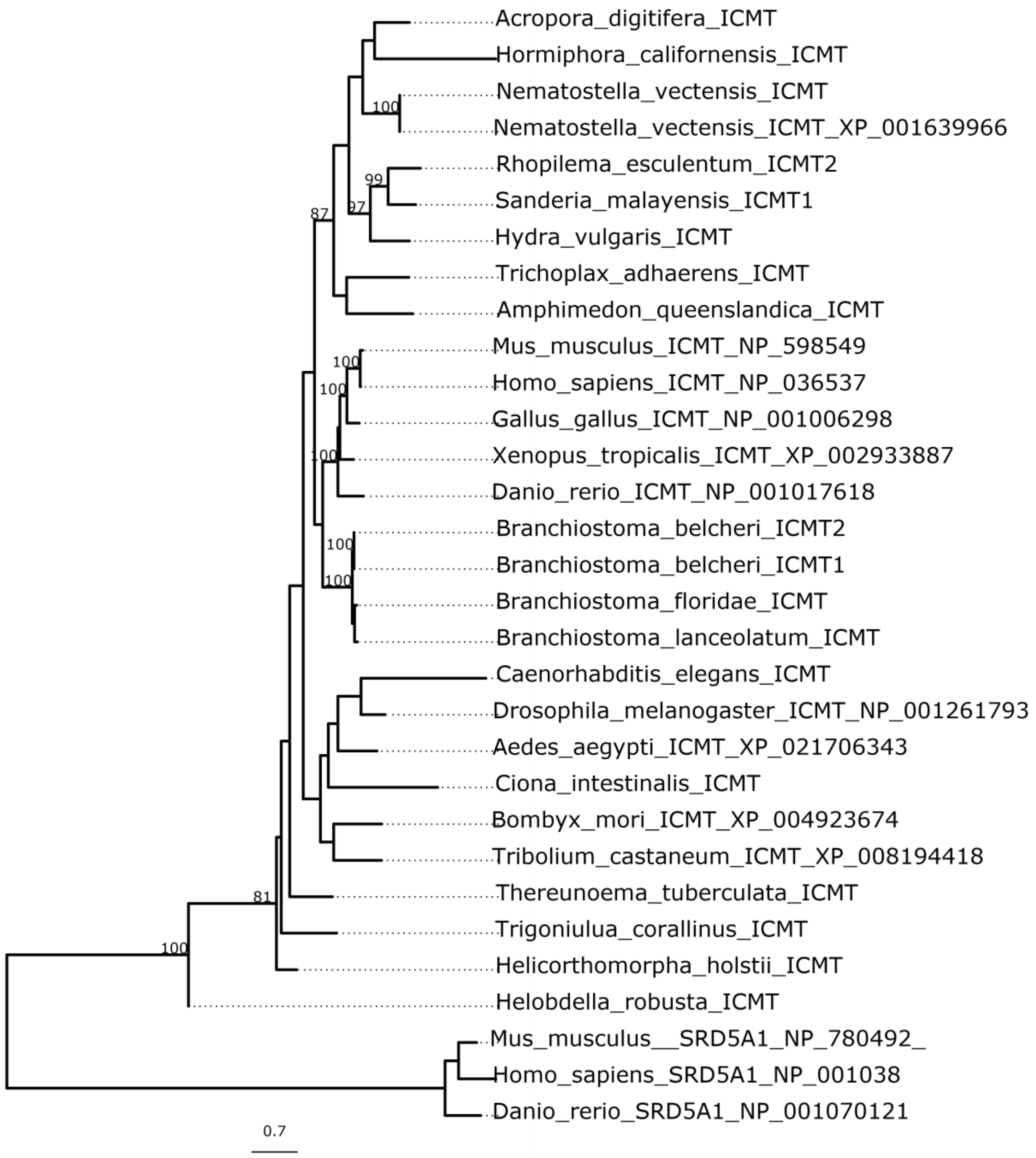

Supplementary Figure S10. Phylogenetic tree of protein-S-isoprenylcysteine O-methyltransferase (ICMT). Tree is constructed with the maximum likelihood (ML) method (LG+F+G4) with 1000 bootstrap replicates. Only bootstrap values larger than 80% are indicated for clarity. Tree rooted using metazoan steroid 5 alpha-reductase 1 (SRD5A1).

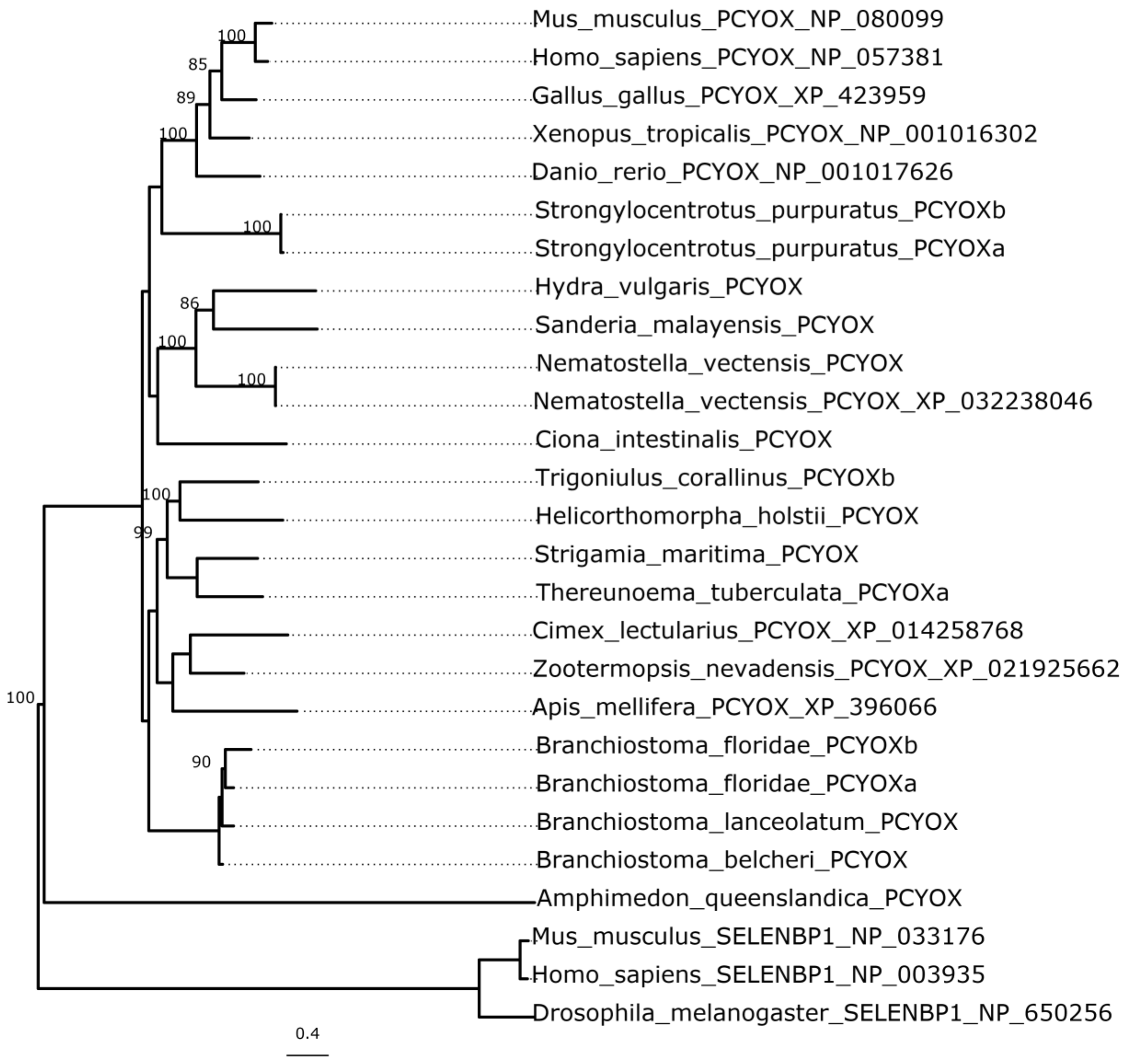

Supplementary Figure S11. Phylogenetic tree of prenylcysteine oxidase (PCYOX). Tree is constructed with the maximum likelihood (ML) method (LG+G4) with 1000 bootstrap replicates. Only bootstrap values larger than 80% are indicated for clarity. Tree rooted using metazoan selenium binding protein 1 (SELENBP1).

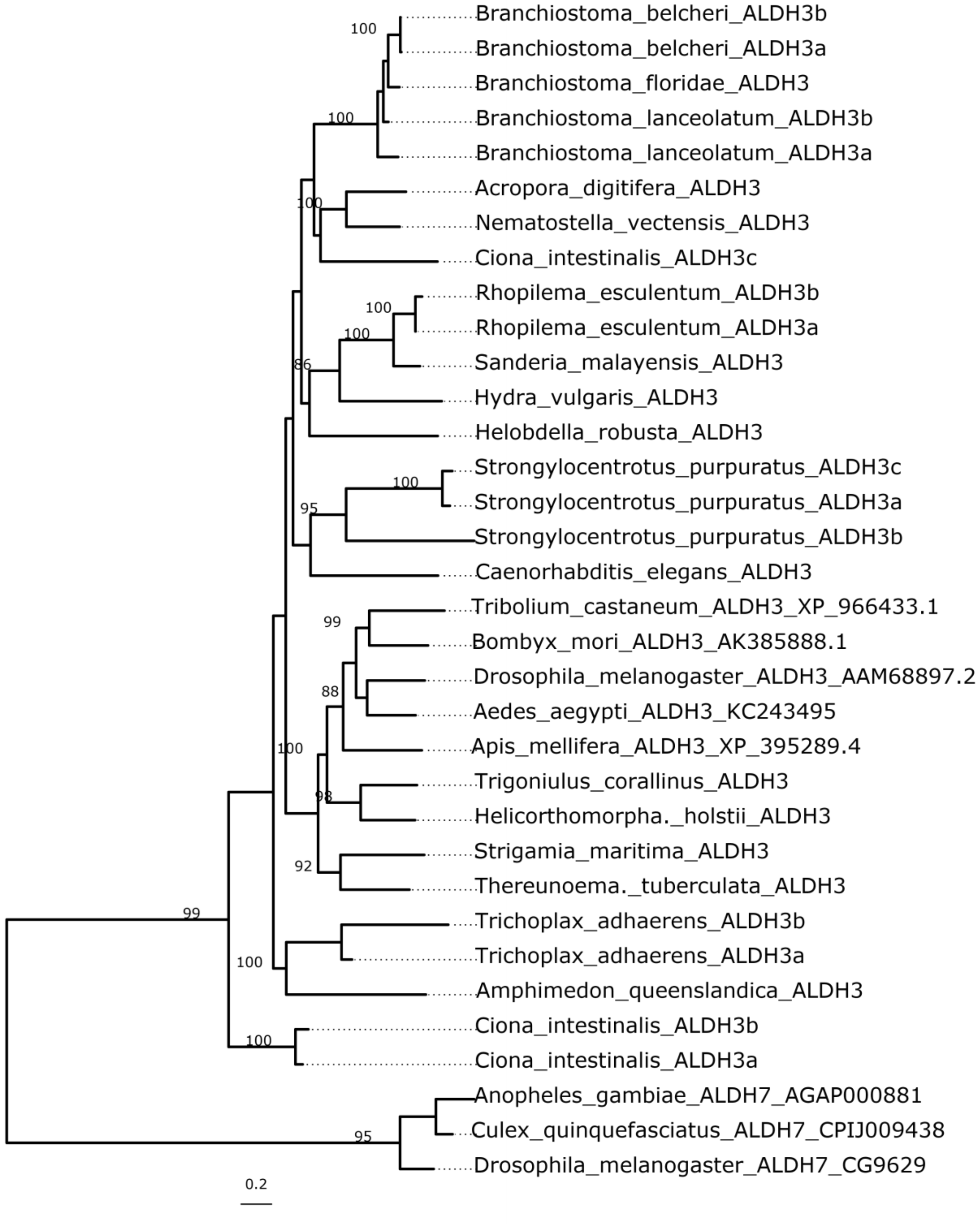

Supplementary Figure S12. Phylogenetic tree of aldehyde dehydrogenase 3 (ALDHIII). Tree is constructed with the maximum likelihood (ML) method with 1000 bootstrap replicates. Only bootstrap values larger than 80% are indicated for clarity. Tree rooted using arthropod aldehyde dehydrogenase 7 (ALDH7).
